# Modulation of network centrality and gray matter microstructure using multi‐session brain stimulation and memory training

**DOI:** 10.1002/hbm.25857

**Published:** 2022-04-04

**Authors:** Friederike Thams, Nadine Külzow, Agnes Flöel, Daria Antonenko

**Affiliations:** ^1^ Department of Neurology Universitätsmedizin Greifswald Greifswald Germany; ^2^ Charité – Universitätsmedizin Berlin, Freie Universität Berlin, Humboldt‐Universität zu Berlin and Berlin Institute of Health, Neurocure Cluster of Excellence Berlin Germany; ^3^ Neurological Rehabilitation Clinic Kliniken Beelitz GmbH Beelitz Germany; ^4^ German Centre for Neurodegenerative Diseases (DZNE) Standort Greifswald Greifswald Germany

**Keywords:** diffusion tensor imaging, eigenvector centrality mapping, graph analysis, object‐location memory, older adults, resting‐state functional connectivity

## Abstract

Neural mechanisms of behavioral improvement induced by repeated transcranial direct current stimulation (tDCS) combined with cognitive training are yet unclear. Previously, we reported behavioral effects of a 3‐day visuospatial memory training with concurrent anodal tDCS over the right temporoparietal cortex in older adults. To investigate intervention‐induced neural alterations we here used functional magnetic resonance imaging (fMRI) and diffusion tensor imaging (DTI) datasets available from 35 participants of this previous study, acquired before and after the intervention. To delineate changes in whole‐brain functional network architecture, we employed eigenvector centrality mapping. Gray matter alterations were analyzed using DTI‐derived mean diffusivity (MD). Network centrality in the bilateral posterior temporooccipital cortex was reduced after anodal compared to sham stimulation. This focal effect is indicative of decreased functional connectivity of the brain region underneath the anodal electrode and its left‐hemispheric homolog with other “relevant” (i.e., highly connected) brain regions, thereby providing evidence for reorganizational processes within the brain's network architecture. Examining local MD changes in these clusters, an interaction between stimulation condition and training success indicated a decrease of MD in the right (stimulated) temporooccipital cluster in individuals who showed superior behavioral training benefits. Using a data‐driven whole‐brain network approach, we provide evidence for targeted neuromodulatory effects of a combined tDCS‐and‐training intervention. We show for the first time that gray matter alterations of microstructure (assessed by DTI‐derived MD) may be involved in tDCS‐enhanced cognitive training. Increased knowledge on how combined interventions modulate neural networks in older adults, will help the development of specific therapeutic interventions against age‐associated cognitive decline.

## Graphical abstract

Previously, we reported behavioral effects of a three‐day visuospatial memory training with concurrent anodal transcranial direct current stimulation (tDCS) over the right temporoparietal cortex in older adults. To investigate intervention‐induced neural alterations we here used functional magnetic resonance imaging (fMRI) and diffusion tensor imaging (DTI) to delineate changes in whole‐brain functional network architecture and underlying microstructural grey matter alterations. We provide evidence for targeted neuromodulatory effects of a combined tDCS‐and‐training intervention, which may help the development of specific therapeutic interventions against age‐associated cognitive decline.

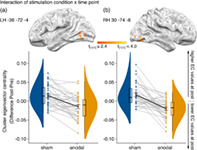

## 1 INTRODUCTION

Transcranial direct current stimulation (tDCS), especially when applied over multiple sessions and combined with training interventions, has emerged as a promising means to modulate cognitive functions in younger adults, older adults, and clinical populations (Berryhill & Martin, 2018; Goldthorpe et al., 2020; Polania et al., 2018). However, neural mechanisms underlying such improvements are poorly understood (Berryhill & Martin, 2018; Horne et al., 2020).

Implementation of neuroimaging to understand specific neural effects of tDCS interventions is of high relevance (Esmaeilpour et al., 2020; Venkatakrishnan & Sandrini, 2012). Especially multi‐modal imaging may be essential to obtain information on the underlying neurophysiological processes and to characterize neuromodulatory tDCS effects (Bergmann et al., 2016). Evidence from studies assessing functional magnetic resonance imaging (fMRI) during or immediately after single‐session application of anodal tDCS over task‐relevant brain areas suggests that the effects are not spatially limited to the stimulated region, but rather unfold on the network level (Keeser et al., 2011; Meinzer et al., 2012), which offers the possibility of modulating an entire functional system. In fact, studies that probed task‐related neural activity during application of anodal tDCS over functionally relevant brain regions have shown modulations on the network level (Holland et al., 2016; Martin et al., 2017). Investigation of changes in resting‐state networks after a single session of either anodal or bilateral tDCS over task‐relevant brain areas further demonstrated its potential to modulate functional connectivity (Meinzer et al., 2013; Sehm et al., 2012; Šimko et al., 2021). Indeed, anodal tDCS may induce cortical excitability changes in activated networks, as demonstrated in studies assessing transcranial magnetic stimulation‐evoked potentials using electroencephalography (Pellicciari et al., 2013; Pisoni et al., 2018; Romero Lauro et al., 2014). At the same time, evidence for functional specific modulation of cognitive functions has been under debate: “conventional” tDCS montages with large electrodes have been criticized for exerting only unspecific effects, because induced electric fields are distributed over a large amount of the cortical surface (Caulfield et al., 2020; Datta et al., 2008; Kuo et al., 2013; Opitz et al., 2015). Nonetheless, the combination of tDCS and cognitive training might result in such specific effects, as it has been suggested that tDCS might support and/or facilitate ongoing activity in already activated networks (i.e., activated by task execution; Monte‐Silva et al., 2013; Nissim et al., 2019). However, so far, only few studies assessed alterations in task‐relevant functional networks after multiple sessions of anodal tDCS and cognitive training (Antonenko, Külzow, De Sousa, et al., 2018; Möller et al., 2017).

Thus, while there is some evidence from single‐session tDCS studies (Meinzer et al., 2013; Pisoni et al., 2018; Sehm et al., 2012; Šimko et al., 2021), and first evidence from multi‐session tDCS studies (Antonenko, Külzow, De Sousa, et al., 2018; Möller et al., 2017) for functional network effects in human participants, whether microstructural plasticity changes accompany tDCS plus training‐related interventions is not fully understood. This knowledge on microstructural plasticity after multi‐session tDCS is crucial, however, given the potential of these approaches to induce long‐lasting neural plasticity (cf., Abellaneda‐Pérez et al., 2019). Evidence from animal studies suggests that alterations of gray matter microstructure, which present an important neurophysiological correlate of learning (Blumenfeld‐Katzir et al., 2011; Perez‐Alvarez et al., 2014; Theodosis et al., 2008), substantially contribute to neuroplastic processes. Recent studies in humans implemented diffusion tensor imaging (DTI) of gray matter mean diffusivity (MD), a measure sensitive to quantify microstructural neuroplasticity, and could for the first time show microstructural correlates of learning in cortical and subcortical regions on a short time‐scale (Brodt et al., 2018; Hofstetter et al., 2017; Sagi et al., 2012). Considering this evidence, it is conceivable that tDCS‐induced plasticity could similarly be detected by investigation of gray matter microstructural changes.

In the current study, we used multi‐modal magnetic resonance imaging (MRI) data from our previous study combining tDCS and visuo‐spatial memory training performance in healthy older adults and older patients with mild cognitive impairment (MCI), acquired immediately before and after the intervention. Behavioral data were published before, showing a beneficial effect of anodal tDCS over sham, particularly for initially low‐performing participants (de Sousa et al., 2020). The ability to link, encode, and recall objects and corresponding locations is highly relevant to everyday life, but, at the same time, this ability is especially prone to age‐associated deterioration (Iachini et al., 2009; Klencklen et al., 2012). We therefore now aimed to delineate specific neuromodulatory effects of this combined intervention, comparing anodal and sham stimulation conditions. We used a graph‐theoretical whole‐brain approach for resting‐state functional MRI data, as predefining networks of interest might not uncover entirely the effects of the intervention. The chosen eigenvector centrality mapping (ECM) approach allows for assumption‐free analysis of functional network connectivity (Chase et al., 2020; Meinzer et al., 2015). Graph theoretical approaches have previously been reported to detect network correlates of cognitive functions in health and disease, and modulations of these functions induced by transcranial electrical stimulation (Antonenko, Nierhaus, Meinzer, et al., 2018; Arnemann et al., 2015; Binnewijzend et al., 2014; Cao et al., 2020; Gundlach et al., 2020; Lou et al., 2015; Meinzer et al., 2012; Meinzer et al., 2013; Schoonheim et al., 2014; Sehm et al., 2012). Interestingly, tDCS‐induced increase as well as decrease in network centrality has previously been associated with improved behavioral performance (Meinzer et al., 2012; Meinzer et al., 2015; Sehm et al., 2012). Thus, the directionality of network effects, that is, whether strengthened connectivity or functional decoupling of a certain region is beneficial for behavioral performance, likely depends on the function under study, its underlying network architecture, and possibly also the study cohort (for example, young versus older adults) (Perceval et al., 2016). In addition to resting‐state networks derived from fMRI, we explored gray matter microstructure using DTI‐derived MD. MD has previously been described as a “nonspecific, but sensitive marker of tissue microstructure” (Assaf, 2018), most likely influenced neurophysiologically by several factors of tissue density such as remodeling of glia cells or changes in neuronal size and shape (Blumenfeld‐Katzir et al., 2011; Sagi et al., 2012). In addition, decreased MD has been shown to reflect rapid learning‐related changes in gray matter, thereby providing an opportunity to measure cortical plasticity alterations (Brodt et al., 2018; Hofstetter et al., 2017; Sagi et al., 2012).

## 2 MATERIALS AND METHODS

Data reported here were obtained during two interventional trials with identical study designs, one including healthy older adults (NCT02110056, https://clinicaltrials.gov/show/NCT02110056) and one including patients with MCI (NCT02110043, https://clinicaltrials.gov/show/NCT02110043). Both trials were approved by the ethics committee of the Charité‐Universitätsmedizin Berlin, Germany and conducted between 2014 and 2017 in accordance with the Helsinki Declaration. All participants gave written informed consent before participation.

**FIGURE 1:**
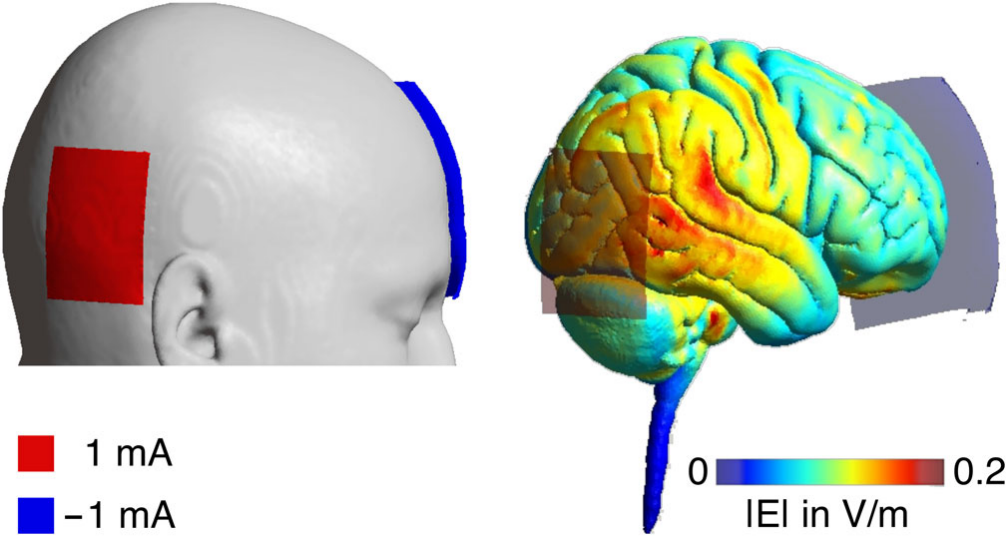
Illustration of electrode placement and electric field distribution of the applied brain stimulation on an MNI head/brain, simulated using SimNibs (Saturnino et al., 2019; Thielscher et al., 2015) for active electrode (anode, red) over the right temporoparietal cortex (T6, size: 7 × 5 cm^2^, 1 mA) and return electrode (cathode, blue) over the contralateral supraorbital region (Fp2, size: 10 × 10 cm^2^, −1 mA). |*E*| below the active electrode: ~0.15 V/m

MRI was acquired 1 day before and after the tDCS‐plus‐training intervention; behavioral results were previously published (de Sousa et al., 2020). Out of 47 participants completing the behavioral intervention, complete MRI datasets from all four time points were available of 35 older adults (8 older adults with MCI and 27 older adults without cognitive impairment) and included in the present article. Sample characteristics are shown in Table 1. Of note, Antonenko et al. (2018) used a subset of the MRI data for a different (between‐subject) analysis: specifically, out of 140 MRI scans included here (i.e., four time points of 35 participants), 30 resting‐state scans of 15 participants (i.e., the first two time points) have been analyzed in the previous publication from our group. In the present study, data from healthy older adults and patients with MCI were pooled for analysis, as there were only few MCI patients with complete imaging datasets. Data were acquired in a counter‐balanced, placebo‐controlled cross‐over, single‐blind study design. Participants underwent two blocks of testing with an interval of 3 months in‐between to prevent carry‐over effects. During each block, participants performed a visuospatial memory training task (Flöel et al., 2012; Külzow et al., 2014) with either concurrent anodal tDCS or sham stimulation on three consecutive days. During training, participants were presented with object‐location pairings on a two‐dimensional street map. Over the course of five learning blocks on each training day, participants had to memorize 30 object‐location pairings. Buildings (i.e., objects) were shown in different locations on the map. Correct locations of the buildings were shown twice within each learning block, whereas incorrect pairings (i.e., the building appearing at a certain “wrong” location) only occurred once during the whole training. Participants indicated correct or incorrect object‐location pairing via button press on a response pad (“yes” or “no”). Primary behavioral outcome, i.e., training success, was computed as the difference of percent correct of the fifth learning block on training day 3 and percent correct of the first learning block on day 1 (de Sousa et al., 2020). Direct current stimulation (neuroConn DCStimulator Plus; neuroCare Group GmbH, Munich, Germany) was delivered via sponge electrodes over the right temporoparietal cortex (anode, centered over T6, 10‐20 EEG system, size: 7 × 5 cm^2^, current density = 0.028 mA/cm^2^) and the contralateral supraorbital cortex (cathode, Fp2, 10‐20 EEG system, size: 10 × 10 cm^2^, current density = 0.01 mA/cm^2^, the large size of the return electrode rendering its current density functionally inert (Nitsche et al., 2007)). Stimulation was delivered with 1‐ mA intensity for 20 min (30 s for sham) with 10 s ramp‐up/down. For further information on the intervention, see de Sousa et al. (2020). We simulated the electric field distribution of the applied stimulation to demonstrate that the target area received a considerable amount of current by performing computational modeling analyses on a standard brain (MNI) with SimNibs (Saturnino et al., 2019; Thielscher et al., 2015; Windhoff et al., 2013) (Figure 1).

**TABLE 1:** Characteristics of the study sample

	*N* = 35
*n* male/female	15/20
*n* with/without mild cognitive impairment	8/27
Age in years	68.0 (6.4)
Education in years^a^	15.2 (2.9)
Beck's depression inventory (Beck et al., 2001)	4.3 (4.2)
Multiple‐choice vocabulary intelligence test (Lehrl, 1977) (max. 37)	32.1 (2.9)

*Note*: Data are shown as the mean (SD) or *n*.

^a^ n/a for *n* = 2.

One day before and one day after the training, MRI was acquired at the Berlin Center for Advanced Neuroimaging with a 3T Siemens Trio MRI system using a 12‐channel head coil. The scanning protocol comprised a high‐resolution T1‐weighted magnetization prepared rapid gradient echo sequence (repetition time = 1900 ms, echo time = 2.52 ms, 192 slices, voxel size = 1 × 1 × 1 mm^3^, flip angle = 9°), a resting‐state fMRI echo‐planar imaging sequence (repetition time = 2300 ms, echo time = 30 ms, 35 slices, voxel size = 3 × 3 × 4 mm^3^, no gap, interleaved acquisition, field of view = 192 × 192 mm^2^, 150 volumes, flip angle = 90°) and a diffusion‐weighted spin‐echo echo‐planar imaging sequence (repetition time = 7500 ms, echo time = 86 ms, 61 axial slices, voxel size = 2.3 × 2.3 × 2.3 mm^3^, 64 directions, *b*‐value of 1000 s/mm^2^, 1 b0). During resting‐state acquisition, participants were instructed not to fall asleep or think of anything in particular. Whether eyes open or closed during resting‐states leads to more reliable network measures is still a matter of debate (Patriat et al., 2013; Van Dijk et al., 2012; Yuan et al., 2014). Importantly, all participants were instructed to keep their eyes closed, to ensure consistency between participants. Preprocessing of resting‐state fMRI data was conducted using Statistical Parametric Mapping, SPM12 (Wellcome Department of Imaging Neuroscience, London, UK; www.fil.ion.ucl.ac.uk/spm/) and CONN toolbox (Nieto‐Castanon, 2020; Whitfield‐Gabrieli & Nieto‐Castanon, 2012). Preprocessing entailed slice timing correction, head motion correction, co‐registration to individual structural T1 images, spatial normalization, spatial smoothing (full width half maximum 6‐mm isotropic Gaussian kernel) and temporal filtering (high‐pass at 100 s = 0.01 Hz). Sources of noise were estimated in the resting‐state data within white matter and cerebrospinal fluid masks using the CompCor method (Behzadi et al., 2007). Nuisance regression included the first five principal components from the CompCor analysis and six head motion parameters obtained from preprocessing (cf., Antonenko, Nierhaus, Meinzer, et al., 2018; Long et al., 2016). Excessive head motion was defined as follows: global mean signal intensity exceeded 5 standard deviations or slice‐to‐slice movement exceeded 0.9 mm, corresponding to the intermediate motion thresholds in the CONN toolbox (Whitfield‐Gabrieli & Nieto‐Castanon, 2012). As part of the default denoising pipeline, noise components for identified outlier scans (exceeding the motion thresholds) are regressed from the blood oxygenation level dependent signal (Power et al., 2014). Resting‐state sequences from pre‐ and post‐training were preprocessed separately and then simultaneously included in the group ECM analysis. Of note, ECM analyses represent a graph‐theoretical data‐driven approach and no prior hypotheses are needed (Bonacich, 2007). An advantage of eigenvector centrality (EC) in comparison to other centrality measures, such as degree centrality, is its ability to represent the entire architecture of the network of interest (Bonacich, 2007). ECM not only accounts for direct connections, but can be used to identify “hubs” or “nodes” that are of high importance or prominence in a network (Lohmann et al., 2010; Wink et al., 2012), similar to Google's “PageRank” algorithm (cf., Wink et al., 2012). Other approaches for assessment of functional network connectivity, such as Independent Component Analysis (ICA; Smith et al., 2009), require a priori decisions, for example on the number of components as well as the network of interest (such as default mode network (DMN) or executive control network, among others). We thus chose ECM as the optimal approach to measure centrality on the whole‐brain level not requiring a priori assumptions while forgoing the high computational demands of other graph analytic methods (Joyce et al., 2010; Lohmann et al., 2010; Wink et al., 2012). ECM analyses using the fastECM software (Wink et al., 2012) were carried out to estimate voxel‐wise eigenvector centralities (i.e., the relevance of each voxel in the whole brain network). Individual EC maps were then entered into a flexible factorial model as implemented in SPM12 (Ashburner et al., 2014), to analyze the interaction of stimulation condition and time point with object‐location memory (OLM) training success as covariate.

To explore potential underlying microstructural mechanisms of functional connectivity modulation observed in the present study, our analysis of gray matter MD was guided by the results of the whole‐brain ECM analysis. To obtain MD of gray matter regions of interest (ROIs), namely the resulting clusters from ECM analysis, diffusion‐weighted images were preprocessed using the Oxford Centre for Functional MRI of the Brain (FMRIB) software library (FSL v.6.0.0, www.fmrib.ox.ac.uk/fsl) and the FSL diffusion toolbox (FDT) including eddy current and subject motion correction, brain extraction and diffusion tensor fitting to obtain individual fractional anisotropy (FA) and MD maps. Individual MD maps were registered to standard space using first linear, then nonlinear transformation. ROI masks were then used to extract individual MD values. MD represents the overall diffusion of water molecules indicated by the mean amount of diffusion in each direction of the diffusion tensor (Johansen‐Berg & Behrens, 2013). Further analysis entailed two linear mixed models for change (post − pre) in left and right ROI MD, respectively, including stimulation condition as factor, OLM training success as covariate, and a condition by training success interaction term. Statistical analyses were carried out with IBM SPSS Statistics 25 (IBM Corp., Armonk, NY) and R software (version 3.6.3, https://www.R-project.org).

## 3 RESULTS

**FIGURE 2:**
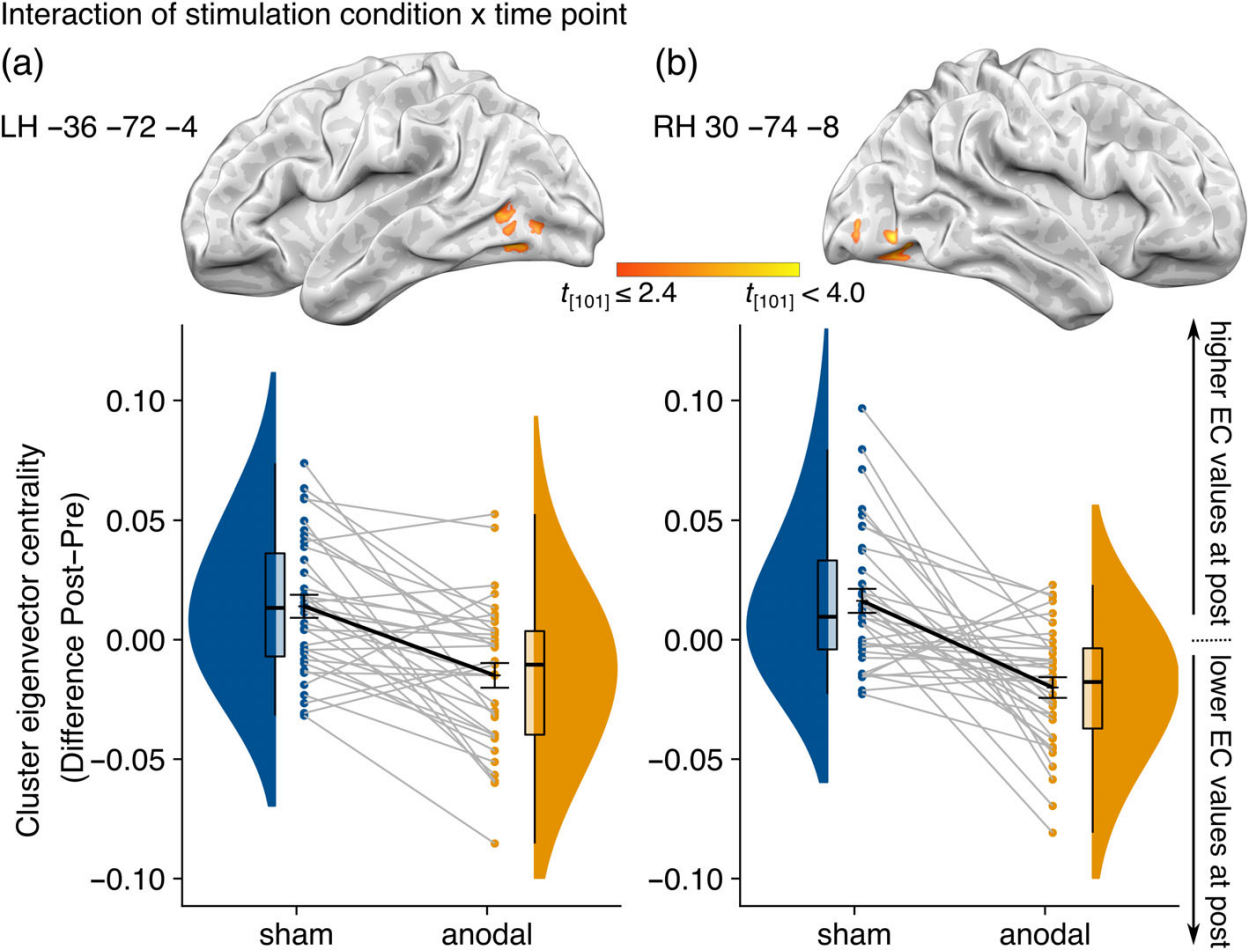
Eigenvector centrality (EC) after three‐day training‐plus‐brain stimulation intervention. Raincloud plots depicting the difference in eigenvector centrality (post − pre) for significant clusters in the left (a) and right (b) hemisphere for anodal and sham groups. For both clusters, EC values after stimulation were decreased in the anodal group compared to sham. Dots represent individual subject data, with lines connecting the respective subject data in each group. Bold black lines represent overall mean and standard error of the mean as error bars. Cluster location is visualized over each plot. Significance thresholds were *p* <.05 FWE correction at the cluster level, cluster‐forming threshold, *p* <.001 uncorrected. LH, left hemisphere. RH, right hemisphere

Analyses of EC revealed a significant interaction of time point and stimulation condition for two bilateral clusters in the temporooccipital cortex (peak coordinates in the lateral occipital complex, LOC, *p* <.05 FWE correction at the cluster level, cluster‐forming threshold, *p* <.001 uncorrected, Figure 2, Table 2), indicating differences in modulation between tDCS and sham conditions. Specifically, EC values after stimulation were decreased in the anodal group compared to sham. Including training success as covariate did not change resultant clusters, thus suggesting no influence of behavioral performance variations on the reported clusters. Similarly, inclusion of order of stimulation conditions as covariate (i.e., first block anodal, second block sham stimulation, or vice versa) did not influence the results. To further explore the effect, we analyzed main effects of time point in both, anodal and sham stimulation conditions separately. This exploration did not yield any significant results.

**TABLE 2:** Regions for eigenvector centrality mapping value interaction of stimulation condition and time point

Region	Cluster size	Peak *X*	Peak *Y*	Peak *Z*	Peak *z* scores
Left lateral occipital complex	97	−36	−72	−4	4.26
Right lateral occipital complex	100	30	−74	−8	4.19

*Note*: *p* <.05 FWE correction at the cluster level. Cluster‐forming threshold, *p* <.001 uncorrected.

**FIGURE 3:**
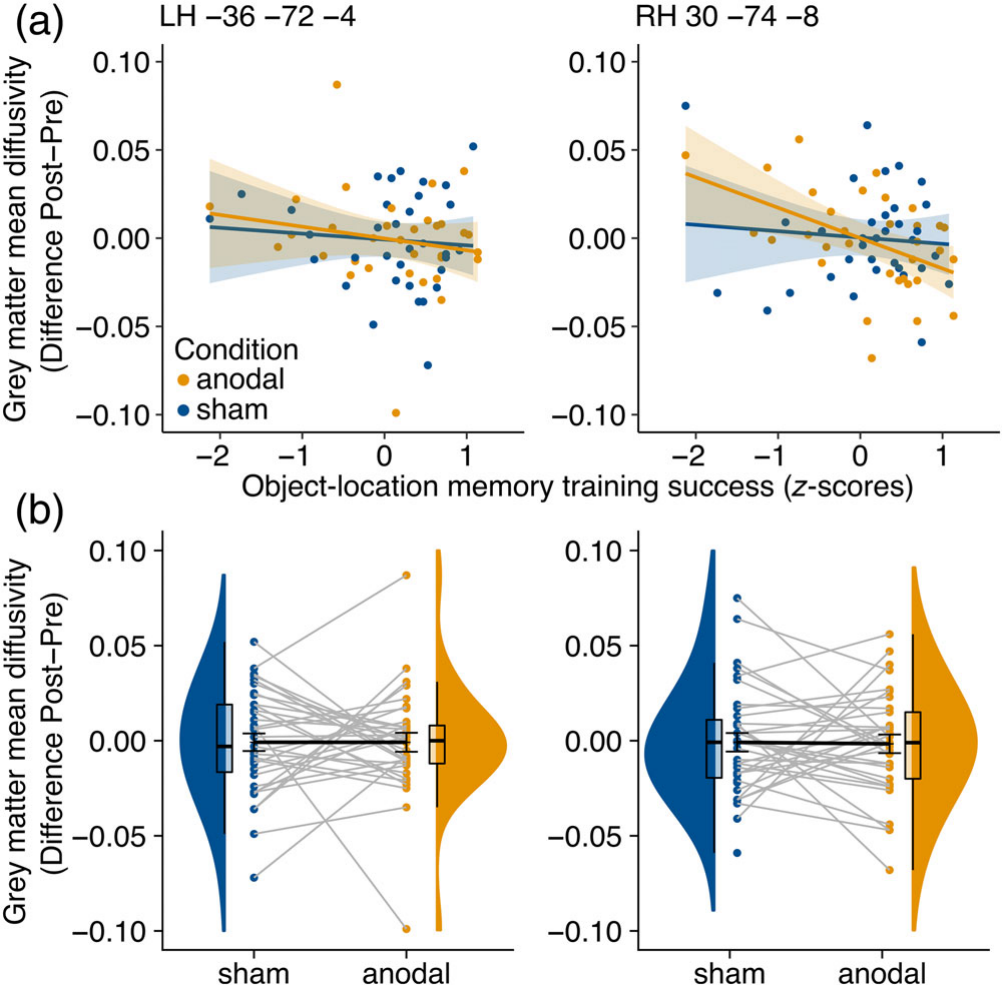
Gray matter mean diffusivity (MD) of left and right lateral temporooccipital cortex clusters that were found in the eigenvector centrality mapping analysis. (a) Significant condition x training success interaction for the right cluster, indicating an association of higher training success and reduced MD after anodal (*r* = −.481, *p* = .005) but not after sham stimulation (*r* = .089, *p* = .610). (b) No significant difference in gray matter MD between anodal and sham conditions. Dots represent individual subject data. LH, left hemisphere. RH, right hemisphere. anodal, anodal tDCS condition; sham, sham tDCS condition

Turning to gray matter MD changes, an interaction between stimulation condition and OLM training success was observed for right LOC (*F*_(1,77.24)_ = 10.85, *p* = .001, Figure 3a, Table 3). This association was likely not driven by correlations between MD at pre assessment and training success (for anodal and sham group, respectively: Pearson's *r* <.1, *p* >.3). Explorative analysis of the interaction by marginal means revealed that decreased MD after stimulation was associated with higher training success in the anodal, but not in the sham condition (effect of stimulation condition [anodal vs. sham] at 25th percentile of training success: 0.8 × 10^−5^, 95% CI [−2.0 × 10^−5^, 0.4 × 10^−5^], *p* = .190; at 75th percentile of training success: −1.3 × 10^−5^, 95% CI [−2.6 × 10^−5^, 2.8 × 10^−8^], *p* = .050). To further illustrate the direction of this interaction, we performed Pearson's correlation analyses and bias estimation for the correlations using bootstrapping (Liu, 2021) for each stimulation condition separately (anodal: Pearson's *r* = −.481, *p* = .005; sham: Pearson's *r* = .089, *p* = .610; bias corrected coefficients: anodal: *r*_corrected_ = −.489, 95% CI [−0.713; −0.175]; sham: *r*_corrected_ = .099; 95% CI [−0.242, 0.418]). Of note, decreased MD values represent lower diffusivity, that is, indicating greater tissue density (Pierpaoli & Basser, 1996). Linear mixed models revealed no effects for the left LOC (all *p* >.246). No differences in gray matter MD changes emerged between conditions in any of the clusters (left LOC: *F*_(1,66.84)_ = 0.013, *p* = .910; right LOC: *F*_(1,56.44)_ = 0.002, *p* = .967, Figure 3b).

**TABLE 3:** Parameter estimates of two linear mixed models for change (post − pre) in left and right ROI MD

Dependent variable	Independent variables	Estimate	*T*	*p*	95% confidence interval
Left lateral occipital complex MD (post − pre)	Condition (sham − anodal)	−7.7	−0.1	.910	−1.4, 1.3
	Training success	−6.7	−1.1	.295	−1.9, 6.0
	Condition × training success	4.5	0.6	.551	−1.1, 2.0
	Intercept	−1.8	−0.04	.972	−1.0, 9.7
Right lateral occipital complex MD (post − pre)	Condition (sham − anodal)	−2.3	−0.04	.967	−1.1, 1.1
	Training success	−2.0	−3.4	<.001	−3.1, −8.1
	Condition × training success	2.1	3.3	.001	3.3, 8.2
	Intercept	−5.1	−0.1	.912	−9.7, 8.7

## 4 DISCUSSION

We here demonstrated for the first time that a 3‐day visuospatial memory training combined with concurrent brain stimulation elicits changes in EC in a hypothesis‐free, data driven approach. Graph theoretical analyses revealed increased functional network segregation of bilateral temporooccipital regions after anodal compared to sham stimulation. Additional explorative analysis of gray matter MD in areas indicated by the graph‐based approach suggests microstructural tDCS‐induced plasticity after successful training.

Our finding of altered interconnectedness of bilateral temporooccipital regions after anodal compared to sham tDCS over right temporoparietal cortex, indicates that multi‐session OLM training with concurrent brain stimulation elicits changes in network centrality (Lohmann et al., 2010; Wink et al., 2012). Of note, as we did not observe a main effect of time point for anodal nor sham stimulation condition, we cannot infer increases or decreases within separate stimulation conditions, but only conclude different modulations of functional connectivity patterns through anodal compared to sham stimulation condition (Bergmann & Hartwigsen, 2021). The observed connectivity modulation suggests a functional decoupling of the stimulated brain area and its left‐hemispheric homolog from the whole brain network. An increased segregation of the LOC, which has been implicated in object perception and object‐location processing (Gillis et al., 2016; Grill‐Spector et al., 2001; Malach et al., 1995), in our cohort of older adults, may reflect a focal mode of action in relevant areas after stimulation (Geerligs et al., 2015; Grady et al., 2016). As reduced specificity of brain networks, that is, reduced segregation between functional networks, is a characteristic of older brains (Betzel et al., 2014; Geerligs et al., 2015; Reuter‐Lorenz & Park, 2014), this result may point toward a reduction of age‐related effects induced by the intervention. In line with this conclusion, previous evidence including young and older adults has suggested the potential of brain stimulation to alter the nonbeneficial consequences of aging on the neural level (Indahlastari et al., 2021; Meinzer et al., 2013, 2015; Reinhart & Nguyen, 2019). However, as we did not include a group of young adults we can only speculate about the age‐dependency of the effect in the present study. In the previous publication of behavioral results, we did not find a main effect of training‐plus‐tDCS intervention on behavior. The intervention only resulted in a small behavioral improvement in the MCI group (de Sousa et al., 2020). However, we argue that analysis of intervention‐related imaging effects, even in the absence of direct behavioral correlates, contributes to the mechanistic understanding of combined cognitive training and tDCS effects (Bergmann & Hartwigsen, 2021). One could speculate that imaging might detect changes on the network level that do not (yet) translate into behavioral changes. However, network changes might indicate for which functions or individuals a given intervention could be helpful, and future studies might then build on these findings and optimize the intervention to yield behaviorally meaningful effects.

Moreover, this finding provides evidence for specific effects in stimulated brain areas of “conventional” tDCS electrode montages when combined with cognitive tasks, even though they induce electric fields that are distributed over large parts of the cortex (Kuo et al., 2013). Thus, ongoing task‐specific brain activation, induced by task execution, interacts with the effects of tDCS and results in network modulation that cannot be explained by either tDCS or task execution alone (Bikson & Rahman, 2013; Monte‐Silva et al., 2013; Nissim et al., 2019; Passow et al., 2017; Pisoni et al., 2018). This finding may also have implications for the interpretation of results derived from electrical field modeling studies (Hartwigsen et al., 2015; Thielscher et al., 2015), as they do not account for the interplay between functional tDCS effects and neural activations. As our results were obtained in an older sample, the age‐specific neurophysiology of the brain has to be considered when interpreting our findings (Perceval et al., 2016). For example, older compared to younger brains, undergo functional and structural network reorganizational processes and changes in neurotransmitter activity (Grady, 2012; Gutchess, 2014). Moreover, structural alterations such as atrophy of gray matter result in increased cerebrospinal fluid (Fjell et al., 2009; Raz et al., 2010). Consequently, the current flow of the stimulation may differ as a function of age‐related altered neurophysiology (Indahlastari et al., 2020; Laakso et al., 2015; Mahdavi et al., 2018; Opitz et al., 2015). Future work will have to determine the generalizability of the combined intervention in terms of age‐specificity of the suggested mechanisms of action (Habich et al., 2020).

Furthermore, while previous studies delineated changes in network centrality measures during (single‐session) application of anodal tDCS over task‐relevant areas (Antonenko, Nierhaus, Meinzer, et al., 2018; Meinzer et al., 2012; Polania et al., 2011; Sehm et al., 2012), the effects of combined multi‐session tDCS‐plus‐training interventions on functional connectivity are yet insufficiently understood. In a previous study (using a subsample of the present data, but in a between‐subject design), we explored ICA‐based resting‐state fMRI connectivity alteration using the same 3‐day visuospatial memory training with concurrent anodal tDCS over the right temporoparietal region as in the present study (Antonenko, Külzow, Sousa, et al., 2018). We found increased connectivity strength within the task‐relevant DMN and between the stimulation target and a central DMN node after the intervention for the anodal tDCS compared to the sham group, in both young and older adults (Antonenko, Külzow, Sousa, et al., 2018). Our current finding extends these observations by demonstrating targeted functional network centrality alterations in the region underneath the anodal electrode and its left‐hemispheric homolog. Of note, we used fMRI to assess the intervention‐induced alterations during resting‐state. Resting‐state fMRI is an established method to capture intrinsic network connectivity, especially enabling the assessment of lasting effects after the intervention (Antonenko, Külzow, De Sousa, et al., 2018; Fox & Raichle, 2007; Möller et al., 2017). Future studies may add to our findings by assessing the effects of combined tDCS and cognitive training during the intervention. Specifically, task‐based fMRI could be used to explore the online effects that may be specifically elucidated by the interaction of brain stimulation during task execution (Esmaeilpour et al., 2020; Li et al., 2019).

We then asked whether microstructural alterations occurred in the regions that were functionally modulated through anodal tDCS. This idea was derived from animal studies and recent evidence in humans, which showed that in‐vivo structural plasticity, as induced by learning, can be observed through diffusivity changes in gray matter (Blumenfeld‐Katzir et al., 2011; Brodt et al., 2018; Hofstetter & Assaf, 2017; Sagi et al., 2012). Previous work has mostly implemented volumetric measures using T1‐weighted anatomical scans to demonstrate learning‐related structural plasticity changes (with or without tDCS) (Allman et al., 2016; Draganski et al., 2004; Engvig et al., 2010; Lövdén et al., 2012; Lövdén et al., 2013; Mak et al., 2017; Taubert et al., 2011). Whether tDCS can induce gray matter plasticity as measured by DTI‐derived MD has not been investigated so far. Our finding of microstructural gray matter changes in the right LOC in association with behavioral training success after anodal tDCS over OLM‐relevant brain regions, suggests responsiveness of the functionally altered area to the intervention. Specifically, our results indicate that tDCS results in reduced MD, pointing toward microstructural plasticity, but only in participants who experience behavioral improvement through the intervention. Underlying mechanisms on the physiological level may be changes in tissue density due to altered neuronal morphology, for example, altered size and shape of axons, dendrites or cell bodies (Blumenfeld‐Katzir et al., 2011). Another supposed physiological mechanism of MD change is altered motility of glial cells, especially astrocytes, which comprise a relevant element in synaptic transmissions and regulation of ion concentrations in the extracellular space (Perez‐Alvarez et al., 2014; Theodosis et al., 2008). Of note, previous studies have suggested an effect of tDCS on the neuropil, for example, on glia cells (Liu et al., 2018). In fact, modulation of the glial membrane potential has been proposed to underlie tDCS effects (Ruohonen & Karhu, 2012), which has been supported by evidence in mice (Monai et al., 2016). Changes of membrane potentials in turn may influence the balance of neurotransmitters and thereby affect systemwide neural communication processes (Ruohonen & Karhu, 2012). Taken together, alterations in MD are most likely caused by multiple factors on the physiological level and we can only speculate about the exact neurophysiological mechanisms (Assaf, 2018). Our results, albeit exploratory, provide novel evidence that learning‐related synaptic plasticity, that is, gray matter microstructural alterations, may be induced by anodal tDCS and cognitive training in individuals who benefit behaviorally from the intervention.

In the current study, we pooled data of healthy older adults and patients with MCI. Previous work has shown EC differences between healthy older adults and patients with MCI (in temporal lobe [Qiu et al., 2016]). In our study, however, we did not find a priori group differences in EC values between healthy older adults and patients with MCI (data not shown). Thus, we are confident that our approach did not constrain interpretability of the results and was thus justified to improve sensitivity of analyses due to otherwise small sample sizes. Nonetheless, future work should include large enough sample sizes to perform group comparisons regarding possible differences in functional and structural modulations through combined training‐plus‐tDCS interventions.

## 5 CONCLUSION AND OUTLOOK

In conclusion, this study contributes to understanding the mode of action of tDCS and cognitive training on functional neural network and microstructural level. We show that a multi‐session tDCS‐plus‐training intervention can lead to more efficient processing on the functional network level. Moreover, we demonstrate for the first time that gray matter microstructural plasticity may be involved in tDCS‐supported cognitive training. Our findings may be specific to visuospatial functions of older adults and future research will have to delineate the effects in other cognitive domains and participant cohorts.

Combined interventions of tDCS and cognitive training present a promising means to counteract cognitive impairments in healthy as well as in pathological aging (Berryhill & Martin, 2018; de Sousa et al., 2020; Goldthorpe et al., 2020). Improved understanding of targeted neuronal network effects of combined interventions in older adults will therefore inform the development of specific therapeutic interventions against age‐associated cognitive decline.

## CONFLICT OF INTEREST

The authors declare no potential conflict of interest.

## AUTHOR CONTRIBUTIONS

*Conceptualization*: Friederike Thams, Agnes Flöel, and Daria Antonenko. *Data acquisition*: Nadine Külzow. *Methodology*, *Investigation*, *Formal Analyses*: Friederike Thams and Daria Antonenko. *Writing – Original Draft*: Friederike Thams and Daria Antonenko. *Writing – Review & Editing*: Friederike Thams, Nadine Külzow, Agnes Flöel, and Daria Antonenko. *Visualization*: Friederike Thams. *Supervision*: Agnes Flöel and Daria Antonenko. *Funding acquisition*: Agnes Flöel.

## Data Availability

The data that support the findings of this study are available from the corresponding authors upon reasonable request.
